# FBXW7 in Cancer: What Has Been Unraveled Thus Far?

**DOI:** 10.3390/cancers11020246

**Published:** 2019-02-19

**Authors:** Bethsebie Lalduhsaki Sailo, Kishore Banik, Sosmitha Girisa, Devivasha Bordoloi, Lu Fan, Clarissa Esmeralda Halim, Hong Wang, Alan Prem Kumar, Dali Zheng, Xinliang Mao, Gautam Sethi, Ajaikumar Bahulayan Kunnumakkara

**Affiliations:** 1 Cancer Biology Laboratory and DBT-AIST International Laboratory for Advanced Biomedicine (DAILAB), Department of Biosciences and Bioengineering, Indian Institute of Technology Guwahati, Guwahati, Assam 781039, India; bethsailo@gmail.com (B.L.S.); kishore.banik@iitg.ernet.in (K.B.); sosmi176106101@iitg.ac.in (S.G.); devivasha@iitg.ac.in (D.B.); 2 Department of Pharmacology, Yong Loo Lin School of Medicine, National University of Singapore, Singapore 117 600, Singapore; phcfanl@nus.edu.sg (L.F.); phsceh@nus.edu.sg (C.E.H.); snrwh@nus.edu.sg (H.W.); csiapk@nus.edu.sg (A.P.K.); 3 Cancer Science Institute of Singapore, National University of Singapore, Singapore 117 600, Singapore; 4 Medical Science Cluster, Yong Loo Lin School of Medicine, National University of Singapore, Singapore 117 600, Singapore; 5 Curtin Medical School, Faculty of Health Sciences, Curtin University, Perth, WA 6845, Australia; 6 The School of Stomatology, Fujian Medical University, 1 Xue Yuan Road, University Town, Fuzhou 350108, Fujian, China; dalizheng@hotmail.com; 7 Institute of Clinical Pharmacology, Guangzhou University of Chinese Medicine, 12 Jichang Road, Baiyun District, Guangzhou 510405, Guangdong, China; xinliangmao@suda.edu.cn; 8 Department of Pharmacology, College of Pharmaceutical Sciences, Soochow University, 199 Ren’ai Road, Suzhou 215123, Jiangsu, China

**Keywords:** FBXW7, SCF complex, tumor suppressor, mutations, cancer, chemoresistance, chemosensitization

## Abstract

The FBXW7 (F-box with 7 tandem WD40) protein encoded by the gene *FBXW7* is one of the crucial components of ubiquitin ligase called Skp1-Cullin1-F-box (SCF) complex that aids in the degradation of many oncoproteins via the ubiquitin-proteasome system (UPS) thus regulating cellular growth. FBXW7 is considered as a potent tumor suppressor as most of its target substrates can function as potential growth promoters, including c-Myc, Notch, cyclin E, c-JUN, and KLF5. Its regulators include p53, C/EBP-δ, Numb, microRNAs, Pin 1, Hes-5, BMI1, Ebp2. Mounting evidence has indicated the involvement of aberrant expression of FBXW7 for tumorigenesis. Moreover, numerous studies have also shown its role in cancer cell chemosensitization, thereby demonstrating the importance of FBXW7 in the development of curative cancer therapy. This comprehensive review emphasizes on the targets, functions, regulators and expression of FBXW7 in different cancers and its involvement in sensitizing cancer cells to chemotherapeutic drugs.

## 1. Introduction

Despite the advancement in the management of cancer, it remains the leading cause of mortality, accounting for 14.1 million new cases and 8.2 million deaths in 2012 worldwide [1,2,3,4,5]. It is well proven that tumorigenesis involves perturbation of various essential molecular pathways and biological processes including ubiquitination. Ubiquitination involves proteasomal degradation via the ubiquitin-proteasome system (UPS) and is the key eukaryotic proteolytic mechanism for more than 80% of proteins involved in cell cycle, cell growth and apoptosis [6]. Thus, dysregulation of the UPS may lead to the development of various diseases including cancer. During ubiquitination, the ubiquitin protein binds with the target protein and necessitates the sequential function of three enzymes namely, ubiquitin-activating enzyme (E1), ubiquitin-conjugating enzyme (E2) and ubiquitin ligase (E3). The ubiquitin ligase (E3) binds to the substrate proteins and subsequently causes their degradation via the 26S proteasomes. Studies showed that modulation of ubiquitin ligase E3 function is one the prime factor for initiation and progression of cancer [7,8].

**Figure 1:**
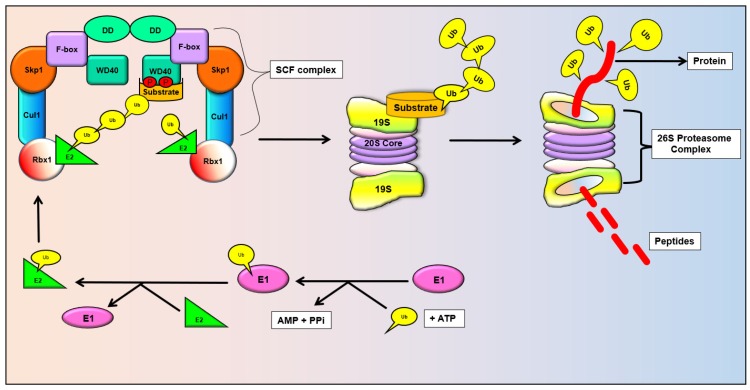
Mode of function of FBXW7.

Among the many types of E3 ubiquitin ligases, the SCF (Skp1-Cullin1-F-box) complex that consists of scaffold protein Cullin1 (Cul1), RING finger protein Rbx1, linker protein S phase kinase-associated protein 1 (Skp1), and F-box protein has been extensively studied [8]. SCF complex/E3 ubiquitin ligase governs the substrate proteins for ubiquitylation and subsequent proteasomal degradation via the UPS (Figure 1). Nearly 80–90% of intracellular proteins are degraded via UPS [9]. The Cul1 domain of SCF functions as the catalytic core, Skp1 domain joins the F-box with Cul1 and Rbx1 domain is essential for the catalytic function of SCF complex [10,11]. The F-box domain can serve as a substrate receptor of the SCF complex and recruits substrate for ubiquitination [8].

In humans, 69 F-box proteins have been identified so far and the F-box motif is usually located in the amino-terminal of the protein and the carboxyl terminal is coupled with other motifs such as WD (tryptophan and aspartic acid) or leucine-rich repeats (LRR). Based on this, F-box is classified as FBXW (F-box coupled with WD repeats), FBXL (F-box coupled with LRRs) and FBXO (F-box with no motifs) [8,12]. The F-box motif of the FBXW and FBXL binds with the Skp-1 subunit while the WD and LRRs motifs recognize large arrays of protein for ubiquitination and undergo proteolysis via the 26S proteasomes [12,13]. Increasing lines of evidence have revealed that aberrant expression of F-box is associated with development, proliferation, angiogenesis, and metastasis of various malignancies [8]. Amongst the F-box family, F-box with 7 tandem WD40 repeats (FBXW7) which are responsible for recognizing and targeting various oncogenic substrates for ubiquitin-mediated degradation has been found to be deregulated in diverse cancers [13,14].

## 2. FBXW7

An F-box with 7 tandem WD40 repeats (FBXW7; also known as Fbw7, Sel-10, hCdc4, hAgo or Archipelago) encoded by the gene *FBXW7*, is a member of F-box, FBXW sub-family with highly conserved F-box motif of about 40 amino-acid [15,16]. It was first identified in budding yeast in 1973 as Cdc4 [17]. The human *FBXW7* gene is located at chromosome 4q31q.3, which is a region deleted in 30% of cancers [18,19], and consists of 4 untranslated and 13 coding exons [20].

**Figure 2:**
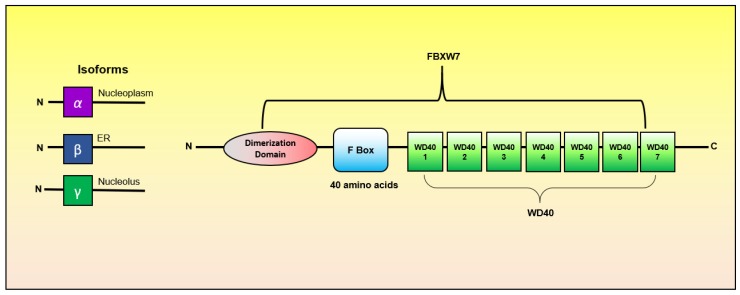
Structure of FBXW7.

The FBXW7 exist in three isoforms namely FBXW7*α*, FBXW7β, and FBXW7γ, with distinct cellular localization, i.e., FBXW7α is located in the nucleoplasm, FBXW7β resides in the endoplasmic reticulum, and FBXW7γ is found in the nucleolus [13]. Structurally, these isoforms differ only at their N-terminal region and share conserved domains in the C-terminal region. Each of them consists of three vital domains, namely (i) dimerization domain, (ii) the F-box domain and (iii) 7 tandemWD40 repeats (Figure 2). The F-box domain binds the FBXW7 to the Skp1 component of the SCF complex [13]. The WD40 repeat makes up the propeller and consists of three arginine residues and binds to the phosphorylated substrate through recognition of conserved phosphorylated domain, called Cdc4 phosphodegron (CPD) phosphorylated by glycogen synthase kinase 3 (GSK3) [16,21]. The dimerization domain helps in the binding of FBXW7 to the substrate [13].

Aforementioned, FBXW7 is one of the key components of the SCF complex, and aids in substrate recognition for ubiquitination and subsequent proteasomal degradation by the 26S proteasome [16]. The ubiquitin-conjugating enzyme (Ubc) attaches to the SCF complex and transports ubiquitin (Ub) into substrates bounded on the F-box protein. After multiple ubiquitin molecules bind to the substrate, it gets degraded by the 26S proteasome [12]. Studies have shown that under normal biological conditions, FBXW7 is essential for safeguarding bone marrow erythroid cells maturation by regulating the expression of cyclin E [22]. It also mediates the differentiation and proliferation of stem and progenitor cells and is involved in maintaining normal hematopoiesis [23]. Moreover, FBXW7 helps in the regulation of NSC (neural stem cell) differentiation and studies have indicated that brain-specific ablation of FBXW7 led to the accumulation of Notch1 as well as c-Jun that may result in increased self-renewal capabilities of NSC. FBXW7 may also be responsible for pluripotency of embryonic stem cells (ESCs) by controlling c-Myc protein stability [16].

More importantly, FBXW7 is considered as a strong p53-dependent tumor suppressor governing human cell cycle progression, cell growth, and tumor development by directing certain oncoproteins such as cyclin E, notch, c-Jun, c-Myc, mammalian target of rapamycin (mTOR), for ubiquitin-mediated proteolysis [24,25,26,27,28,29]. Additionally, numerous studies have indicated that inactivation or downregulation of FBXW7 can result in the deregulation of these oncoproteins and may lead to tumorigenesis in human as well as the development of chemoresistance [6,30].

## 3. Substrates of FBXW7

**Figure 3:**
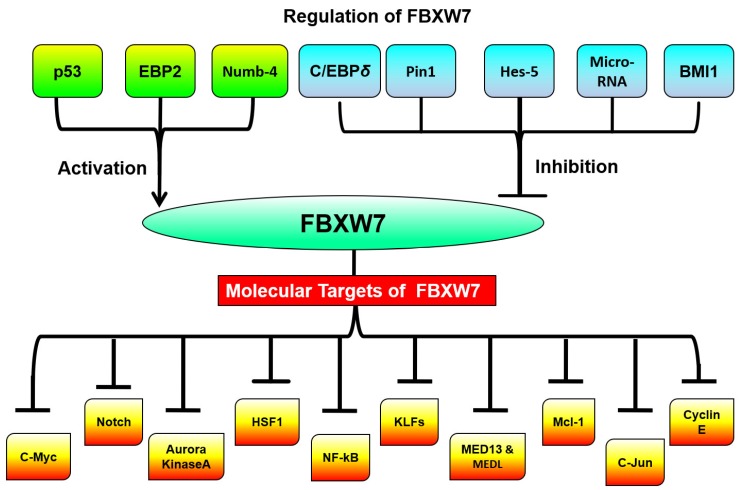
Regulators and targets of FBXW7 in cancer.

As indicated above, FBXW7 is a potent tumor suppressor and that can regulate the expression level of many oncoproteins that partake in cellular pathways by directing them for proteasomal degradation thus preventing tumorigenesis. For instance, it is well evidenced that deregulation of proto-oncogene c-Myc leads to the development of many human cancers. It has been reported that FBXW7α ubiquitylates c-Myc in the nucleoplasm and FBXW7γ ubiquitylates c-Myc in the nucleolus, thereby facilitating proteasomal degradation and thus inhibiting the c-Myc ability to promote cancer cell growth [11,13]. However, downregulation of FBXW7 can lead to an increased level of both cellular and active chromatin-bound c-Myc, without affecting other FBXW7 targeted genes [31] (Figure 3).

Another oncoprotein, Notch, that has been reported to participate in the development of tumor can also be ubiquitylated by FBXW7 for degradation [32]. However, in a T-cell acute lymphoblastic leukemia (T-ALL) patient, a mutation in FBXW7 caused the production of an elevated amount of Notch protein [33]. Further, deletion of FBXW7 in bone-marrow-derived stromal cells (BMSCs) resulted in the accumulation of Notch which consequently rendered elevation of chemokine (C-C motif) ligand 2 (CCL2), thereby promoting cancer metastasis in vivo [34].

Additionally, it is well-known that transcription factor, nuclear factor kappa B (NF-κB) controls cell survival, tumor invasion, and drug resistance, through the regulation of multiple oncogenic gene products [35,36,37,38,39,40,41,42,43,44,45,46,47,48,49,50,51,52,53,54,55,56,57,58,59,60,61,62]. Reports have indicated that NFκB2/p100 can interact with FBXW7 thereby prompting its degradation in a GSK3β phosphorylation-dependent manner [63,64]. Another substrate, heat-shock factor 1 (HSF1), that regulates the heat-shock response and supports malignancy has been reported to be ubiquitylated by FBXW7α via a conserved motif phosphorylated by GSK3β and ERK1. In most cancers, including melanoma, FBXW7α is mutated or downregulated resulting in impaired degradation of HSF1, which increased the accumulation HSF1, thus, enhancing the metastatic potential of human melanoma [65].

Another substrate of FBXW7 is a transcription factor, kruppel-like factor2 (KLF2), has been reported to exert cell growth-inhibitory, pro-apoptotic and anti-angiogenic activities [28,66]. Studies have revealed that FBXW7 may mediate KLF2 degradation via phosphorylation of KLF2 by glycogen synthase kinase-3 (GSK3) at the two CPD domain on KLF2 [28]. In line with this finding, it has been demonstrated that knockdown of FBXW7 can upregulate KLF2 in endothelial cells thereby regulating the endothelial cell migration, angiogenesis, and endothelial barrier integrity in vivo [67]. FBXW7 has also been reported to ubiquitylate and degrade oncogenic transcription c-Jun through the phosphorylation of c-Jun by GSK3β [68]. Besides these, FBXW7 can also ubiquitylate several other essential proteins such as Aurora A, cyclin E, mTOR (mechanistic target of rapamycin), SREBP (sterol regulatory element-binding protein), NF1 (Neurofibromatosis type 1), NRF1 (nuclear factor E2-related factor 1), and MED13 (mediator 13) [28,31].

## 4. Regulation of FBXW7

Accumulating studies have shown that mutations and/or deletions of FBXW7 have been implicated in numerous human tumors, thus, indicating that aberrant regulation of FBXW7 is one of the prime factors for tumorigenesis. Some of the regulators of FBXW7 includes tumor suppressor p53, Pin1 (Peptidyl-prolyl cis-trans isomerase NIMA-interacting1), C/EBP-δ (CCAAT/enhancer-binding protein-δ), Hes-5 (Hairy and Enhancer-of-split homologues 5), Numb, and microRNAs (miRNAs) such as miR-27, and miR-223 [28] (Figure 3).

### 4.1. p53

Studies have found that the initial exon of FBXW7 harbors p53-binding site, making it one of the targets of p53 [69]. Moreover, reports have revealed a significant upregulation of FBXW7 when p53 deficient cells were infected with adenovirus-mediated wild-type p53. It was also reported that the expression level of FBXW7 may directly correlate with that of p53. More importantly, p53-dependent suppression of FBXW7 activated aurora-A, c-Jun, and Notch leading to genetic instability [28]. Studies have also indicated that in gastric cancer patients with the p53 mutation, decreased expression of FBXW7 with distinctively poor prognosis was observed [25]. Further, it has been shown that FBXW7 and p53 cooperatively inhibited advanced and chromosomally unstable intestinal cancer. Moreover, FBXW7-mutated colorectal cancer cells showed inhibition of phosphorylation of p53 at serine-15. In neuroendocrine cancer, p53 mutation was found to cause inhibition of FBXW7, resulting in upregulation of growth promoter aurora-A [70]. Thus, these studies collectively support p53 as one of the key regulators of FBXW7 and that targeting the p53 signaling pathway would offer a suitable approach to replenish FBXW7 for the development of anti-cancer therapies [28].

### 4.2. C/EBP-δ

The protein CCAAT/enhancer-binding protein-δ (C/EBPδ), a member of the C/EBP transcription factor family that binds to the DNA through its leucine zipper scissors is another regulator of FBXW7 [71]. It acts as a tumor suppressor and participates in the regulation of cell proliferation and apoptosis but has pro-metastatic properties [71,72]. It has been reported that C/EBPδ enhanced mTOR stability by directly inhibiting the expression of FBXW7, resulting in elevation of hypoxia and inflammatory signaling which in turn promoted tumor cell survival [71,73]. Further studies have revealed that C/EBPδ inhibited the expression of FBXW7, thereby increasing the activities of the FBXW7 substrates such as mTOR and HIF-1α and potentiated metastasis in mammary tumors [74].

### 4.3. Pin1

Another studied regulators of FBXW7 is prolyl isomerase Pin1 an enzyme that particularly isomerizes Ser/Thr-Pro peptide bonds after phosphorylation to control their conformational changes [28,75]. It has been reported that it may be overexpressed in a majority of human cancers and is associated with poor prognosis [75]. Reports have revealed that Pin1 can negatively regulate the stability and function of FBXW7 by binding to FBXW7 and inducing self-ubiquitination and degradation by altering their dimerization. Thus, overexpression of Pin1 can significantly downregulate FBXW7, thereby consequently augmenting cancer cell progression [28,76].

### 4.4. Other Regulators

Up-regulation of the Notch signaling effector, Hes-5 is observed in many human cancers and it has been indicated that Hes-5 suppressed the transcription of FBXW7β in colon cancer cells [28,32]. Studies by Jiang et al. depicted that tumor suppressor, Numb4 positively regulate the expression of FBXW7, stimulating its assembly and activation, thus encouraging degradation of its substrate Notch in glioma stem cells [77]. Additionally, it is well demonstrated that aberrant microRNA (miRNA) expression is associated with cancer development. Studies have shown that miR-27 targets FBXW7 and enhanced expression of miR-27 downregulated FBXW7 [78,79]. This downregulation accelerated tumor growth through reduction of FBXW7-mediated ubiquitin-dependent degradation of various oncoproteins including c-Myc, c-Jun, cyclin E1 and Notch 1. Conversely, ablation of miR-27a enhanced FBXW7 expression levels, thereby lowering the levels of its oncogenic substrates such as cyclin E [78,79]. Further, in leukemia, miR-27 was found to be upregulated leading to depletion of FBXW7 [79]. Similarly, in gastric cancer, miR-223 functions an oncogene and found to negatively regulate FBXW7 expression that governs the cellular apoptosis, proliferation, and invasion [80]. In line with this, Kumar et al. demonstrated that the Notch-mediated activation of miR-223 suppressed FBXW7 in T-cell acute lymphoblastic leukemia (T-ALL) [81].

Additionally, it has also been found that miR-32 induced cell proliferation, migration and evaded apoptosis in breast cancer in vitro by downregulating FBXW7 [82]. Similarly, in cervical cancer cells upregulation of miR-92a caused downregulation FBXW7, thereby promoting cell proliferation and invasion [83]. Recently, it has also been found that miR-182 increased the proliferation of non-small cell lung cancer cells by suppressing FBXW7 [84]. Further, NF-κB is another possible regulator of FBXW7. It has been indicated that NF-κB p65 which is upregulated in human bladder cancer cells significantly enhanced the cancer cell migration by stabilizing the activity of FBXW7 which in turn ubiquitylated and induced degradation of RHO guanosine diphosphate dissociation inhibitor (RhoGDI)-α protein. Thus, enhancing the tumor suppressive activity of FBXW7 through its regulators can serve as a novel approach in cancer. Nonetheless, further studies on the regulation of FBXW7 expression is warranted for a clearer understanding of the role of FBXW7 in tumorigenesis.

## 5. Genetic and Epigenetic Alterations of FBXW7 in Cancer

In various human cancers, the purview of the genetic alterations in FBXW7 may foothold its role in tumor suppression. It is in turn responsible for the functional inactivation and degradation of various proto-oncogenes ultimately triggering tumorigenesis [85,86]. Mutation, deletion, and hypermethylation are the main causes for the inactivation of FBXW7 thereby resulting in cancer progression [19,86,87]. Seldom, the FBXW7 is found to be mutated in cancers of the breast, cervix, esophagus, gastric, liver, lung and pancreas [85]. Rather, monoallelic and biallelic *FBXW7* gene deletions or promoter hypermethylation are predominantly observed in different cancers for example bladder, breast and cervical cancer. Missense point mutation of FBXW7, however, is the most common type of genetic alteration which impinges the three critical arginine residues of the β-propeller in its phosphate-binding pockets [88].

The different tumors usually express functional wildtype protein by retaining the second wildtype allele of *FBXW7*. Mono-allelic deletion of FBXW7 displays a milder tumor phenotype compared to the complete gene loss in mouse models [89,90]. Therefore, the FBXW7 mutants are assumed to act as dominant negative alleles, which eventually cause functional inactivation of the wildtype protein [85,88]. The FBXW7 heterozygous mouse displays reduced tumorigenesis compared to knock in mouse harboring a heterozygous FBXW7 mutation in the intestine and the hematopoietic system [88,90,91]. Compared to the FBXW7-null animals, the hematopoietic stem cells in *FBXW7^Mut/+^* mice can exhibit substantial accumulation of Myc but does not display the hyper-proliferative phenotype characteristic of FBXW7-null animals [90].

Several in vitro, in vivo and clinical studies have shown that FBXW7α is ubiquitously expressed and has broad tissue distribution. However, the expression of FBXW7β was found to be differentially expressed in different cell lines and in tissue localization. DNA and histone modifications epigenetically regulate the FBXW7β promoter. It is found to be methylated in 51% of breast cancer tumors and 43% in different cancer cell lines [19]. Hypermethylation of the FBXW7 promoter is often linked with mutations in p53, which results in suppressed FBXW7 expression through increased expression of the DNA methyltransferase 1 (DNMT1). Kitade et al. reported that ovarian cancer patients’ display decreased FBXW7 expression with mutated p53 [92]. Histone modifications also play a critical role in the regulation of FBXW7 expression. Enhancer of zeste homolog 2 polycomb repressive complex 2 (EZH2), a histone methyltransferase helps in addition of three methyl groups onto the histone H3 residue, H3K27me3, of FBXW7 which ultimately leads to silencing of FBXW7 gene function [2].

Augmented expression of Notch target gene and *Hes5* transcriptional repressor causes the suppression of *FBXW7* gene expression and forms a positive feedback loop that strengthens the FBXW7 loss-of-function phenotype [93]. An activated Notch allele induced T-cell leukemia in mice and shows stabilization of Myc, SREBP1 and several other substrates. Further, the reduction of p53 does not ameliorate the disease onset emphasizing the functional difference between complete gene loss and FBXW7 mutants. However, in other tissues of *FBXW7^Mut/+^* mice, most tested FBXW7 substrate level remains unaffected with an exception of TGIF1 and KLF5 implicating that the effect of FBXW7 mutations on substrate turnover is vastly context-dependent [91]. Interestingly, FBXW7 mutation ameliorates *Apc^min^*-driven intestinal tumorigenesis but the adenomas arising in these mice also possess normal levels of Myc, Notch and Jun. Hence, heterozygous FBXW7 mutations may promote tumorigenesis via regulation of “non-canonical” substrates such as TGIF1 and KLF5 [88]. Keeping in mind the indispensable role of FBXW7 in the maintenance of physiological substrate levels, it is, therefore, essential to understand the mechanisms controlling its activity.

## 6. Deregulation of FBXW7 in Different Types of Cancer

It is well established that in cancer, the expressions of various genes involved in cell survival, proliferation, invasion, metastasis, chemoresistance and apoptosis are dysregulated [5,94,95,96,97,98]. Interestingly, FBXW7 also plays an important role in the proteasomal degradation of proteins involved in the regulation of cell proliferation and survival such as c-Myc and cyclin E, thereby causing cell cycle exit (G0 phase). Hence, perturbation of the expression of FBXW7 is considered as one of the major causes of cancer development and progression [25]. It was reported that FBXW7 gene mutation in primary human tumors had an overall frequency of 6% point mutation (nonsense and missense mutation) [15,99,100]. In this point mutation, missense mutation leading to the substitution of key arginine residues (Arg^465^ and Arg^479^) of the WD40 domain takes place, initiating damage of the substrate-binding site of the protein, thereby enhancing tumorigenesis [100]. Besides mutations, FBXW7 pathway may also be perturbed due to the suppression of its expression in tumors or due to the aberration of its regulators [73]. Thus, the normal functioning of FBXW7 may be imperative for the prevention of malignancies [16].

### 6.1. Brain Cancer

In glioblastoma, FBXW7 is reported to be downregulated. Studies showed that targeted inhibition of overexpressed microRNA-10b (miR-10b) in glioblastoma can lower the activity of miRNA-15/16 thereby, suppressing its direct targets including FBXW7. Further, knock-down of FBXW7 amplified the expression of cell cycle proteins including cyclin A2 and cyclin E2 in basic condition and miR-10b deficient glioma cells, affirming its role as a regulator of cell cycle proteins’ degradation [101]. Moreover, p53 mutation was found to contribute to the development of gliomas by enhancing the expression of c-Myc via downregulating FBXW7, thereby protecting against apoptosis caused by c-Myc [102]. Studies have also shown that in human gliomas, expression of FBXW7β mRNA is specifically suppressed and plays a key role in the pathogenesis of glioma with increased expression of CCNE1, Myc, and AURKA [103,104]. In medulloblastoma, FBXW7 is either downregulated or mutated, which increased the expression of SOX-9 that further confers cell migration, metastasis, and chemoresistance [105]. In line with this, in vitro overexpression of FBXW7 in U251 and U373 human glioblastoma cells were found to remarkably inhibit the proliferation, invasion and migration [106]. Studies showed that non-coding RNA, human metastasis-associated lung adenocarcinoma transcript 1 (MALAT1) can exert tumor suppressive action by down-regulation of miR-155. Since, FBXW7 mRNA is a direct target of miR-155 in glioma, downregulating miR-155 by MALATI resulted in an enhanced expression of FBXW7, thereby suppressing the glioma cell proliferation [107]. It has also been well established that circular RNAs (circRNAs) have crucial roles in carcinogenesis. Recently, studies have indicated that FBXW7 circular RNA, circ-FBXW7 has the potential to repress tumorigenesis in brain cancer and may serve as a prognostic marker for glioma [108] (Table 1).

### 6.2. Breast Cancer

In breast cancer, the level of FBXW7 mRNA is comparatively lower than in normal tissues and is associated with poorer prognosis [110,164]. Consistent with this, in vitro silencing of FBXW7 substantially upregulated c-Myc and cyclin E thus, accelerating breast cell proliferation as well as G1-S transition [110]. Additionally, one of the profuse cholesterol metabolites, 27-hydroxycholesterol was found to increase the stability of c-Myc in MCF-7 breast cancer cell via repression of FBXW7 [114]. Further, studies have reported that FBXW7 inhibited breast cell proliferation and tumorigenesis in part by targeting KLF5 for degradation via the ubiquitin-proteasomal pathway [109]. Aforementioned, FBXW7 targets cyclin E for proteolysis. In breast cancer cells, mutated FBXW7 caused significant upregulation of cyclin E, thereby augmenting breast cancer cell proliferation in vitro [165]. Depletion of FBXW7 in triple negative breast cancer (TNBC) was found to upregulate one of the key sensor Egl-9 Family Hypoxia-Inducible Factor 2 (EGLN2), thereby contributing to TNBC development.

Another negative regulator of FBXW7, miR-32 was found to be upregulated in breast cancer cells, and directly binds to the 3′-UTR region of FBXW7 and hence reducing FBXW7 expression which resulted in increased breast cancer cell proliferation, migration and inhibited apoptosis [82]. Studies have affirmed that an oncogene *FAM83D* (family with sequence similarity 83, member D) present on chromosome 20q has a significant role in breast cancer development by downregulating FBXW7 resulting in amplification of its oncogenic substrates such as mTOR [111].

Aforementioned, the C/EBPδ is one of the negative regulators of FBXW7 and is reported to be induced by hypoxia in breast cancer in vitro and in vivo. This induced C/EBPδ can suppress FBXW7 in breast cancer, consequently increasing oncogenic mTOR/AKT/S6K1 signaling [166,167,168,169,170,171,172,173,174,175] as well hypoxia-inducible factor-1α (HIF-1α) required for hypoxia adaptation, thereby promoting tumor metastasis [74]. In vitro forced overexpression of FBXW7 repressed breast cancer cell proliferation and promoted apoptosis by targeting the oncoprotein, metadherin (MTDH) for proteolysis [116] (Table 1).

### 6.3. Colorectal Cancer (CRC)

Colorectal tumor mutation profiling showed a missense mutation of FBXW7 in chromosome number 4 with a change in the amino acid sequence R425C [176]. A missense mutation was correlated with poor overall survival in colorectal cancer (CRC) patients [177]. The FBXW7 mRNA level was found to be considerably lesser in colorectal tumor tissues compared to the corresponding normal tissues. Additionally, reports suggested that CRC patients with low expression of FBXW7 showed a poor prognosis. In vitro studies showed that suppression of FBXW7 increased colorectal cancer cell proliferation by upregulating c-Myc and cyclin E [119]. Furthermore, it has been reported that rapamycin-insensitive companion of mTOR (Rictor), forms a complex with FBXW7 and promote degradation of c-Myc and cyclin E in CRC cells [120]. In vitro and in vivo studies showed aberrant phosphorylation of p53 at serine 15 in human FBXW7-deficient CRC cells. Further, loss of function of FBXW7 accounts for the development of resistance to chemotherapeutic drug oxaliplatin in HCT116 colorectal cancer cells due to a reduced level of phospho-p53(Ser15) [178]. Another study showed that the depletion of FBXW7 in HCT-116 enhanced the expression of the oncogenic protein enolase 1 (ENO1) in CRC in vitro [122]. It has also been reported that downregulation of FBXW7 promoted epithelial-mesenchymal transition (EMT) and metastasis in CRC cells. However, treatment of colorectal cancer cells with mTOR inhibitor, rapamycin effectively reversed the FBXW7-deficient driven EMT and metastatic characteristics of CRC cells [99].

Additionally, the heat shock protein 90 (Hsp90) was found to exert its anti-cancer effects in CRC cells in vitro via FBXW7-dependent degradation of MCL-1 [124]. Studies have also evinced that knockdown of the family with sequence similarity 83, member D (FAM83D)-promoted apoptosis in CRC in vitro by causing upregulation of FBXW7 which consequently degraded Notch1 [125]. Reports have indicated that upregulation of miR-182 and miR-503 transformed colon adenoma to adenocarcinoma by jointly reducing the expression of FBXW7. Conversely, inhibiting the expression of both miR-182 and miR-503 caused up-regulation of FBXW7 in colon cancer AAC1 cells and considerably reduced the tumor size in xenograft models [179]. Recently, miR-92b was found to potentiate CRC cell proliferation, invasion, and migration by suppressing FBXW7 in vitro and in vivo [180]. Polo-like kinase 2 (Plk2) was found to instigate tumor growth and evade apoptosis in CRC cells in vitro and in vivo by degrading FBXW7 which rendered subsequent stabilization of cyclin E [123] (Table 1).

### 6.4. Esophageal Cancer

In esophageal squamous cell carcinoma (ESCC), loss of FBXW7 expression has been indicated due to genetic alteration. Studies showed that cases showing loss of FBXW7 copy number exhibited decreased expression of FBXW7 with a poorer prognosis when compared to cases without loss of copy number [181,182]. As mentioned, FBXW7 is negatively regulated by miR-223 and miR-223 expression was found to be high in ESCC patients which are associated with poor prognosis through the suppression of FBXW7 [28,183]. Additionally, mounting evidence has suggested the role of dysregulated miR-27a-3p in tumorigenesis of various types of cancers. Studies have found that miR-27a-3p is upregulated in ESCC cell lines compared to the corresponding normal cell line and enhanced esophageal cancer cell proliferation by markedly suppressing FBXW7 [127] (Table 1).

### 6.5. Gastric Cancer (GC)

In gastric cancer (GC) mutation of FBXW7 is observed in both initial and advanced stages. Lee et al. reported six hCDC4/FBXW7 somatic mutations in gastric cancer case studied, out of which four were missense mutations, one was frameshift deletion and one comprised of nonsense mutation [184]. Low expression of FBXW7 was observed in primary gastric cancer and contributed to the poor survival and minimal response to adjuvant therapy [132]. Studies revealed that loss of CDC4/FBXW7 promoted amplification of c-Myc in both early-onset of gastric cancers (EOGC) and conventional gastric carcinogenesis [185]. Additionally, Calcagno et al. revealed that downregulation of FBXW7 and subsequent amplification of c-Myc contributed to the aggressiveness of this disease [128] (Table 1).

Recently, it has been shown that FBXW7 haploinsufficiency accelerated gastric carcinogenesis in N-methyl-N-nitrosourea (MNU)-induced GC mice model by triggering DNA damage and upregulation of c-Myc [131]. It has also been indicated that miR-25 was overexpressed in GC tissues and promoted GC progression in vitro via repressing the function of FBXW7 by binding it to the 3’-UTR of FBXW7. Conversely, restoration of FBXW7 remarkably inhibited the proliferation, invasion, and migration of GC cells [129] (Table 1). The overexpression of miR-223 in gastric cancer cells also attenuated the level of FBXW7, thereby increasing the GC cell proliferation, invasion and induced chemoresistance to trastuzumab in vitro [80,186]. Furthermore, in vitro and in vivo overexpression of FBXW7 in GC was found to markedly induce apoptosis and inhibited EMT by binding and degrading Ras homologue gene family, member A (RhoA), thereby manifesting its tumor suppressive action in GC [130].

### 6.6. Gynecological Cancers

FBXW7 has been reported to be mutated in at least 16% of human endometrial tumors. These mutations reside predominantly at the substrate-binding domain or at the amino-terminal region of the protein [20]. Gu et al. reported a missense mutation in SKOV3 ovarian cancer cells line with repressed FBXW7 [104] (Table 1). In cervical cancer, there is a marked upregulation of microRNA miR-92a, which binds to 3’UTR of the FBXW7 and inhibits the expression of FBXW7, thus promoting tumor progression and invasion [83]. When HeLa cervical cancer cells were treated with MAPK pathway inhibitor UO-126, FBXW7 expression was remarkably increased, indicating the involvement of FBXW7 in suppressing HeLa cell proliferation [187].

### 6.7. Hepatocellular Carcinoma (HCC)

In hepatocellular carcinoma (HCC), the mutation frequency of FBXW7 ranges from 27.8% to 50% in a particular case studied [142]. Studies have found downregulation of FBXW7 in HCC tissues compared to their adjacent tumor tissues, which may contribute to increased tumor size, and poor prognosis [143,188]. Administration of recombinant human adenovirus-p53 (rAd-p5\Gendicine) in HCC significantly upregulated FBXW7 and downregulated its substrates c-Myc and cyclin E, resulting in the inhibition of cellular growth and apoptosis in vitro and in vivo [142]. It was further proven that FBXW7 instigated apoptosis in HCC in vitro and in vivo through ubiquitination and degradation of the oncoprotein Yes-associated protein (YAP) [143]. Further, studies have indicated that STAT1 exerted its tumor-suppressive role in HCC by upregulating p53 and FBXW7, which resulted in decreased expression of their downstream targets including cyclin A, cyclin D1, cyclin E, CDK2, Hes-1, and NF-κB p65. [144]. Hence, FBXW7 may serve as a potential target for the treatment of HCC patients [142] (Table 1).

### 6.8. Leukemia

Missense FBXW7 mutation has been reported in T cell acute lymphoblastic leukemia (T-ALL) as well as in B-cell acute lymphocytic leukemia [90,189]. FBXW7 mutation is found in 8–12% of T-ALL patients [190]. T-ALL patients with mutations in FBXW7 produced an elevated amount of Notch1, c-Myc and cyclin E [81,90,190,191,192]. Additionally, oncogenic transcription factor TAL1/SCL is abnormally expressed in T-ALL cells and led to an up-regulation of miR-223, which in turn significantly reduced FBXW7 and eventually conferred a marked increase of its oncogenic clients including c-Myc, Notch1, and cyclin E. [138]. On the contrary, it has been also reported that in primary T-ALL, loss of function of FBXW7 resulted in upregulation of glucocorticoid receptor α (GRα), enhancing glucocorticoids sensitivity. This increase in sensitivity can enhance the glucocorticoid treatment response, providing a favorable prognosis in T-ALL patients [137].

Remarkably, two FBXW7 mutants, D510E and D527G exhibited oncogenic function in the presence of HTLV-I Tax, mutated p53 R276H, or c-Myc F138C in T-cell leukemia (ATL). Studies showed that in ATL cases, mutated FBXW7 acts as an oncogene and holds a crucial role in the pathogenesis of ATL in part by losing their ability to bind to Notch1, thereby resulting in increased Notch1 signaling [139]. The increased NOTCH signaling further imparted resistance to gamma-secretase inhibitors (GSI) in T-ALL cells [133]. Recent studies showed that in ATL cases, the reduced expression of FBXW7 caused an increased expression of c-Myc and was associated with poorer prognosis [193]. Interestingly, a natural diterpenoid, oridonin exhibited its anti-cancer activity in leukemia in vitro and in vivo by promoting the FBXW7-mediated ubiquitin-proteasomal degradation of c-Myc [136]. Further, FBXW7 promoted apoptosis in T-All cells in vitro by ubiquitylating and degrading a pro-survival protein, MCL-1 [135]. Similarly, in activated B-cell like diffuse large B-cell lymphoma (ABC-DLBCL), exogenous overexpression of FBXW7 instigated apoptosis by inducing proteasomal degradation of signal transducer and activator of transcription 3 (STAT3), thus offering a novel approach for the treatment of ABC-DLBCL patients [140] (Table 1).

### 6.9. Lung Cancer

According to the TCGA data analysis, mutation frequency of FBXW7 was found to be 2.2% in 507 lung adenocarcinomas cases studied and 4.7% in lung squamous cell cancer cases studied which remarkably resulted in downregulation of FBXW7 leading to a poorer prognosis of lung cancer patients [86,194]. In support to this, in vitro and in vivo silencing of FBXW7 in non-small cell lung cancer (NSCLC) can significantly trigger EMT, promote migration and invasion and confer resistance to gefitinib treatment [151]. In lung adenocarcinoma patient, mutation of FBXW7 resulted in upregulation of mTOR [195]. Further, it has been revealed that the oncogenic activity of miR-367 is mediated in NSCLC cells by degrading its downstream target FBXW7, which eventually assisted in maintaining the activation of Wnt signaling [148]. Another report has shown that Akt inhibitor, API-1 (4-amino-5,8-dihydro-5-oxo-8-β-D-ribofuranosyl-pyrido[2,3-d]pyrimidine-6-carboxamide) can induce apoptosis in lung cancer cells by triggering FBXW7-mediated degradation of MCL-1 [145]. Additionally, terminal differentiation-induced lncRNA (TINCR) was found to inhibit lung cancer cell proliferation and invasion by sequestering miR-544a from its target FBXW7, which caused an increased upregulation of FBXW7. Moreover, a recent study has indicated that overexpression of FBXW7α mediated oncogenic transcription factor Cys2His2 zinc-finger 322A (ZNF322A) for degradation, thereby inhibiting ZNF322A-induced lung cancer progression in vitro and in vivo [147] (Table 1). Hence, these studies indicate the important function of FBXW7 in inhibiting lung cancer development and progression.

### 6.10. Pancreatic Cancer

In pancreatic adenocarcinoma, it has been found that mutations in FBXW7 at the exons 8 and 9 can induce upregulation of cyclin E [196]. In vitro silencing of FBXW7 significantly enhanced pancreatic cancer (PC) cell proliferation, migration, and invasion and rendered resistance to gemcitabine and nab-paclitaxel due to the accumulation of MCL1 [156] (Table 1). It has been demonstrated that in pancreatic ductal adenocarcinoma (PDAC), the nuclear exporter protein CRM1/Exportin 1/Xpo1 is highly expressed which can inhibit the activity of nuclear FBXW7, thereby enhancing the amount of nuclear Notch1. Studies have found that the anti-cancer activity of specific inhibitors of nuclear export (SINE) such as KPT-185 in Colo-357 PDAC xenografts is partly due to the accumulation of nuclear FBXW7 and subsequent degradation of Notch-1, c-Myc, and VEGF. [154]. Additionally, studies have found that FBXW7 directs β-catenin for its degradation, thereby disrupting Wnt/β-catenin signaling pathway. Due to the downregulation of FBXW7 in PC cells, the Wnt/β-catenin signaling pathway is aberrantly activated leading to the progression of PC. Thus, this study showed that by overexpressing FBXW7, its tumor suppressive action may be executed on PC cell through degradation of β-catenin [155].

### 6.11. Prostate Cancer

In prostate cancer, examining the expression level of FBXW7 is associated with the disease state and recurrence making it an important biomarker for determining the efficacy of proteasome target therapy [197]. In prostatic small cell neuroendocrine carcinoma (SCNC) mutations in p53 induced overexpression of miR-25 and inhibited FBXW7. This inhibition of FBXW7 resulted in upregulation of aurora kinase A which directs cancer cells proliferation and aggressive behavior of prostate SCNC [198].

### 6.12. Renal Cancer

In renal cancer patients, the expression of the FBXW7 was found to be aberrated by a constitutional t(3;4)(q21;q31) and is suspected to partake in the development of renal cell carcinoma (RCC) [24]. In RCC, upregulation of FBXW7 can lower the level of c-Myc and c-Jun, thereby reducing the proliferation rate of renal cancer cells and further prompted apoptosis [158]. Further, upregulation FBXW7 in 86-O and ACHN RCC cells can considerably inhibit metastasis and EMT via downregulating metalloproteinase (MMP)-2, MMP-9, and MMP-13 expression [159] (Table 1). Wilms’ tumor (WT) is one the most prevalent pediatric renal tumor wherein FBXW7 is either mutated or deleted in roughly 4% of tumors examined which resulted in the amplification of its target, Myc family, MYCN [199].

### 6.13. Skin Cancer

In skin cancer, studies found that allele-specific deletion of FBXW7 is observed in 7,12-dimethylbenz[a]anthracene (DMBA)/12-O-tetradecanoylphorbol-13-acetate (TPA) induced skin tumor in mice [17]. An in vivo allele-specific deletion of FBXW7 is considered as the germline modifier of tumor susceptibility [29]. In UV-induced skin cancer, FBXW7α transcripts are reduced, and c-Jun is stabilized. This denotes that deregulation of FBXW7 is involved in the development of skin cancer [17]. Interestingly, Ishikawa et al. reported that FBXW7 may not be simply a tumor suppressor as it was found to exhibit two conflicting roles in skin carcinogenesis, i.e., it may either inhibit or promote tumor formation via degrading c-Myc or Notch respectively [160].

Aydin et al. demonstrated that inactivation of FBXW7 is observed in melanoma cells that causes accumulation of Notch1, thereby promoting angiogenesis [161] (Table 1). Reports have suggested that FBXW7 expression is remarkably lower in primary melanoma than in dysplastic nevi and further lowered in the metastatic state compared to primary melanoma and its reduced expression is associated with poor 5-year survival of melanoma patients. Further, in vitro studies have revealed that FBXW7-inhibited melanoma cell migration via the mitogen-activated protein kinase/extracellular signal-regulated kinase (MAPK/ERK) signaling pathway. Thus, ablation of FBXW7 in melanoma cells leads to increased cell migration and stress fiber formation [15]. Moreover, FBXW7 was also found to regulate the oncogene MITF in melanoma. In vitro silencing of FBXW7 in melanoma considerably enhanced MITF/PGC-1 signaling contributing to the augmentation of mitochondrial transcription program and may result in poor outcomes for the patients [163] (Table 1).

### 6.14. Other Cancers

Studies have shown that in cholangiocarcinoma patients with low expression of FBXW7, upregulation of c-Myc and Ki-67 is observed with larger tumor size compared to those with higher FBXW7 expression [118]. Moreover, deficiency of FBXW7 was found to induce metastasis in cholangiocarcinoma cells via promoting EMT in vitro and in vivo [117]. In intrahepatic cholangiocarcinoma (IHCC), FBXW7 downregulation is directly linked with lymph nodes metastasis and poorer prognosis with 3 years survival rates of 29.4% than those with high expression of FBXW7 with 3 years survival rates of 72.7%. Thus, FBXW7 expression has potential as an independent prognostic marker for cholangiocarcinoma [200]. In the case of cervical cancer, cases with low expression of FBXW7 exhibited poor overall survival [201]. In osteosarcoma tissues, it has been shown that FBXW7 is downregulated compared to normal bone tissues. Moreover, in vitro and in vivo overexpression of FBXW7 in osteosarcoma strikingly instigated apoptosis and inhibited tumor progression via the degradation of c-Myc and cyclin E [27] (Table 1). It has also been elucidated that in bone marrow-derived stromal cells (BMSCs), loss of FBXW7 induced cancer metastasis via upregulation of the chemokine CCL2, which increased the monocytic myeloid-derived suppressor cells (Mo-MDSCs) as well as tumor-associated macrophages (TAMs) [34]. In oral cancer, low expression of FBXW7 was found to be associated with poor prognosis. Further, studies have revealed that miR-24 can potentiate the proliferation and metastasis in human tongue squamous cell carcinoma via inhibiting FBXW7 expression by binding to its 3-UTR [152,153]. Overall, these studies suggest the potential of FBXW7 as a prognostic marker and its importance as a tumor suppressor for the development of novel and effective therapy for cancer patients.

## 7. Role of FBXW7 in Cancer Cell Chemosensitization

Accumulated evidence has suggested that inactivation or downregulation of FBXW7 may lead to the development of chemoresistance in various cancers, such as breast cancer, colorectal cancer, gastric cancer, and non-small cell lung cancer (Table 2). Hence, activation or upregulation of FBXW7 has been indicated to overcome chemoresistance and sensitize the cancer cells to chemotherapies and increased the therapeutic efficacy of the existing treatments. On the contrary, Takeishi et al. suggested that expression of FBXW7 has implications in cancer drug resistance and that ablation of FBXW7 in combination with anti-cancer drugs might be a promising therapeutic strategy for chronic myeloid leukemia (CML) patients. For instance, CML, the leukemia-initiating cells (LICs) remained quiescent due to the reduced level of c-Myc caused by FBXW7 as a result of which the disease often relapses. However, ablation of FBXW7 reduced the quiescence of LIC and enhances their sensitivity to imatinib drug treatment [202].

Further, it is well established that eliminating cancer stem cells (CSCs) is a promising approach for enhancing the efficacy of anticancer agents. Evidently, studies have found that FBXW7 is upregulated in colorectal CSCs which led to the acquirement of chemoresistance. However, in vitro knockdown of upregulated FBXW7 can enhance the efficacy of chemotherapeutic drugs including irinotecan, thus offering a feasible approach for obliterating colorectal CSCs [203].

On the other hand combination of FBXW7 overexpression with chemotherapeutic drug temozolomide notably sensitized the glioblastoma cells to temozolomide via downregulating Aurora B, MCL-1 and Notch-1, thereby signifying its potential as a target for therapy [106]. Moreover, downregulation of FBXW7 in MDA-MB-468R breast cancer cell induced resistance to the drug paclitaxel by the accumulation of its substrates myeloid cell leukemia 1 (MCL-1) and polo-like kinase 1 (PLK1). However, in vitro upregulation of FBXW7 significantly chemosensitized MDA-MB-468R cells to paclitaxel treatment [113]. In HCC, in vitro silencing of FBXW7 was found to impart resistance against doxorubicin, however, induced overexpression of FBXW7 significantly chemosensitized the HCC cells to doxorubicin by suppressing the EMT [204]. In CRC, an increase in the level of FBXW7 expression was directly associated with increased doxorubicin sensitivity in vitro. Moreover, it has also been reported that inactivation of miR-223 in CRC LoVo cells upregulated FBXW7 which enhanced the chemosensitivity of LoVo cells to doxorubicin by reducing the EMT [126]. Similarly, in GC it has been shown that inhibition of miR-223 restored the expression of FBXW7 and enhanced the trastuzumab-induced apoptosis [186]. Further, EMT is one of the prime factors accountable for the initiation of chemoresistance and FBXW7 has been found to notably partake in regulating the EMT in cancer cells [30]. Studies have indicated that non-small cell lung cancer (NSCLC) cells with low expression of FBXW7, such as NCI-H1299 cells, exhibited mesenchymal phenotype and are more resistant to cisplatin than cells with an epithelial phenotype. However, upregulation of FBXW7 markedly enhanced the sensitivity of NSCLC cells to chemotherapeutic drugs including cisplatin via modulation of EMT [205]. Additionally, FBXW7 was found to be downregulated in EGFR inhibitor-resistant NSCLC and reactivation of FBXW7 was found to sensitize the NSCLC cells to targeted therapy by facilitating the degradation of MCL-1 [149]. Interestingly, another study showed that although silencing of FBXW7 in NSCLC mediated taxol resistance, it also enhanced the sensitivity to a class I-specific histone deacetylase (HDAC) inhibitor, MS-275 which then eliminated the taxol resistance [146]. Further, in pancreatic cancer (PC), it has also been demonstrated that upregulation of FBXW7 substantially enhanced the chemosensitivity of the PC cells to gemcitabine via increased expression of equilibrative nucleoside transporter 1 (ENT1) [157]. Additionally, in nasopharyngeal carcinoma (NPC) cells, multidrug resistance-associated protein (MRP) was found to enhance the resistance of FBXW7-deficient NPC cells to cisplatin. Conversely, increased expression of FBXW7 remarkably induced chemosensitivity in these cells by downregulation of MRP [206]. Collectively, the majority of the studies suggest FBXW7 as a chemosensitizer that aids in overcoming chemoresistance for better and effective treatment of cancer patients.

## 8. Conclusions and Future Perspective

FBXW7, a crucial component of ubiquitin ligase SCF complex is a potent tumor suppressor that maintains the expression level of various growth regulator proteins by assisting them to the ubiquitin proteasomal system (UPS) for degradation, thereby preventing unregulated cell growth and tumorigenesis. However, FBXW7 is mutated or its pathway is perturbed in many human malignancies including colorectal, leukemia, breast, brain, ovary, cervical, ovary, endometrial, prostate, and gastric. Recent studies have demarcated the close association between mutation of FBXW7 and cancer progression as well as the role of FBXW7 in chemoresistance, which open up a new standpoint regarding the curative potential of targeted therapy. Hence, detection of the mutation status of the FBXW7 may serve as a suitable diagnostic biomarker and also play an invaluable factor in determining suitable individualized therapy [70,130]. As FBXW7 mutations are often heterozygote, determining the effect of monomeric and dimeric forms of FBXW7 on its mutational status is imperative for further understanding of FBXW7 function [207]. Moreover, in-depth studies must be carried out for elucidating the complex network of FBXW7 and its substrates and regulators which would further cater a clearer understanding of the pathogenesis of cancer and possibly finding out novel targets for effective treatment of cancer [201]. Several studies have indicated that induced overexpression of FBXW7 has the potential to chemosensitize cancer cells to chemotherapies. Nevertheless, studies have also found its implication in chemoresistance, implying the need for further studies. In addition, among the many substrates of FBXW7, the ones specifically involved in inducing chemoresistance has yet to be revealed [30]. Hence, despite the numerous studies on FBXW7, its role in cancer drug resistance remains questionable, recommending further elucidation.

## Figures and Tables

**Table 1:** Role of FBXW7 in few selected cancers.

Cancer	In Vitro/In Vivo/Clinical	Cell Lines	Expression of FBXW7	Modulation of FBXW7	Mechanism of Action	Ref.
Bone	In vitro	U2OS, MG-63	↑FBXW7	Ectopic overexpression	↓c-Myc; ↓cyclin E	[27]
	In vivo	Mouse xenografts	↑FBXW7	Ectopic overexpression	↓c-Myc; ↓cyclin E	[27]
	In vitro	BMSCs	↓FBXW7	-	↑CCL2	[34]
Brain	In vitro	A172, U87MG, U251MG, U373MG	↓FBXW7	-	↑CCNE1; ↑MYC;	[104]
					↑AURKA	
	In vivo	Mouse xenografts	↓FBXW7	Overexpressed p53 mutants (C132W and R270C)	↑c-Myc	[102]
	In vitro	U87, SHG139	↑FBXW7	MALAT1 induced overexpression	↓Cell viability	[107]
	In vitro	U343MG, Daoy	↓FBXW7	siRNA-mediated silencing	↑SOX9	[105]
	In vitro	U251, U373	↑FBXW7	Ectopic overexpression	↓Aurora B; ↓MCL-1;	[106]
					↓Notch1	
	In vitro	U251, U373	↑FBXW7	Ectopic overexpression	↓c-Myc	[108]
Breast	In vitro	DLD1	↑FBXW7	Ectopic overexpression	↓KLF5 protein	[109]
	In vitro	T47D	↓FBXW7	siRNA-mediated silencing	↑c-Myc; ↑cyclin E	[110]
	In vitro	MCF10A, BT549	↓FBXW7	Overexpression of FAM83D	↑mTOR	[111]
	In vivo	*Wnt9b*^+/−^ & *Eya1*^+/−^	↓FBXW7	-	↓Eya1 ubiquitination	[112]
	In vitro	MCF7, T47D, MDA-MB-231	↓FBXW7	siRNA-mediated silencing	↑MCL-l; ↑PLK-1	[113]
	In vitro	MCF-7	↓FBXW7	Suppression by 27-HC at transcriptional level	↑Myc	[114]
	In vitro	MDA-MB-453, MCF-7 and MCF-10A	↓FBXW7	shRNA-mediated silencing	↑EglN2	[115]
	In vitro	MCF-7, MDA-MB-231	↓FBXW7	Binding of miR-32 to the 3-UTR of FBXW7	↓Apoptosis	[82]
	In vitro	MDA-MB-23, SKBR	↑FBXW7	Ectopic overexpression	↓MTDH	[116]
Cholangioc-arcinoma	In vitro	HuCCT1, RBE	↓FBXW7	shRNA-mediated silencing	↑EMT	[117]
	In vivo	HuCCT1-shFBXW7 injected mice	↓FBXW7	shRNA-mediated silencing	↑EMT	[117]
	In vitro	Tissue samples	↓FBXW7	-	↑c-Myc; ↑Ki-67	[118]
Colorectal	In vitro	LoVo, Colo 201	↓FBXW7	siRNA-mediated silencing	↑c-Myc; ↑cyclin E	[119]
	In vitro	SW620, HT29, HCT116	↓FBXW7	Knockdown of Rictor	↑c-Myc; ↑cyclin E	[120]
	In vivo	*FBXW7*^flox/flox^ mice	↓FBXW7	Conditional deletion	↑c-Myc; ↑cyclin E	[121]
	In vitro	HCT116, DLD-1	↓FBXW7	Depletion	↑EMT	[99]
	In vitro	HCT116	↑FBXW7	Ectopic overexpression	↓ENO1	[122]
	In vitro	SW480, RKO	↓FBXW7	Degradation by PLK2	↑Cyclin E	[123]
	In vivo	Mouse xenografts	↓FBXW7	Degradation by PLK2	↑Cyclin E	[123]
	In vitro	HCT116, DLD1, RKO, LoVo	↑FBXW7 mutant	-	↑MCL-1	[124]
	In vitro	SW480, HCT116	↑FBXW7	FAM83D knockdown	↓Notch1	[125]
	In vitro	HT-29, SW480, SW620, LoVo	↓FBXW7	Binding of miR-223 to the 3-UTR of FBXW7	↑EMT	[126]
Esophageal	In vitro	TE8, Eca109, EC9706, KYSE30	↓FBXW7	Binding of miR-27a-3p to the 3-UTR of FBXW7	↓G1/S arrest	[127]
Gastric	In vitro	ACP02, ACP03	↓FBXW7	Deletion of one copy of FBXW7	↑c-Myc	[128]
	In vitro	-	↓FBXW7	Binding of miR-25 to the 3-UTR of FBXW7	-	[129]
	In vitro	AZ-521, MGC-803, BGC-823, SGC-7901	↑FBXW7	Ectopic overexpression	↓RhoA	[130]
	In vivo	Mouse xenografts	↑FBXW7	Ectopic overexpression	↓RhoA	[130]
	In vivo	*FBXW7* knockout mice	↓FBXW7	Haploinsufficiency	↑c-Myc	[131]
	In vitro	Tissue samples	↓FBXW7	-	↓Survival; ↓response	[132]
Leukemia	In vitro	DU528, CEM, Jurkat	↑FBXW7 mutant	Missense mutations of arginine (R465 & R505)	↑MYC; ↑DELTEX1	[133]
	In vitro	Tissue samples	↑FBXW7 mutant	Arginine substitutions at R479, R465, R505, and R689	↑NOTCH1; favorable outcome	[134]
	In vitro	*FBXW7*−/− DLD1	↑FBXW7	Ectopic overexpression	↓MCL-1	[135]
	In vitro	Jurkat, CCRF-CEM	↑FBXW7	Oridonin- mediated upregulation	↓c-Myc	[136]
	In vivo	*FBXW7* knock-in mice	↑FBXW7 mutants	Missense mutation	↑c-Myc stability	[90]
	In vitro	Molt4, K562	↓FBXW7	shRNA-mediated silencing	↑GRα	[137]
	In vivo	T-ALL xenografts	↑FBXW7 mutant	R479Q mutation	↑GR stability	[137]
	In vitro	Jurkat cells	↑FBXW7	Knockdown of TAL1	↓Myc; ↓Notch1;	[138]
					↓Cyclin E	
	In vitro	MT1	↑FBXW7 mutant	Mutation at arginine residues R479Q, R505C, and R465H	↑Notch 1	[139]
	In vitro	SU-DHL-2, OCI-LY-3.	↑FBXW7	Ectopic overexpression	↓STAT3	[140]
	Clinical	50 patients	↑FBXW7 mutant	-	Better clinical outcome	[141]
Liver	In vitro	SMMC-7721, HepG2, Hep3B, Huh7	↑FBXW7	Adenoviral delivery of p53	↓c-Myc; ↓cyclin E	[142]
	In vitro	HepG2, Hep3B	↑FBXW7	Flag-FBXW7 overexpression	↓YAP	[143]
	In vivo	Mouse xenografts	↑FBXW7	Flag-FBXW7 overexpression	↓YAP	[143]
	In vitro	SMMC7721, HepG2	↑FBXW7	STAT1 overexpression	↓Cyclin A, D1, E; ↓CDK2;	[144]
					↓Hes-1; ↓NF-κB p65	
Lung	In vitro	A549, HCT116	↓FBXW7	siRNA-mediated silencing	↑MCL-1	[145]
	In vitro	H2009, H1975	↓FBXW7	siRNA-mediated silencing	↑MCL-1	[146]
	In vitro	H1299, H460	↑FBXW7	-	↓ZNF322A	[147]
	In vivo	Mouse xenografts	↑FBXW7	-	↓ZNF322A	[147]
	In vitro	A549, H460, H1299	↓FBXW7	Binding of miR-367 to the 3-UTR of FBXW7	↑Wnt signaling	[148]
	In vitro	PC-9, HCC827, H3122, H3255, H1975, H1299	↓FBXW7	shRNA-mediated silencing	↑MCL-1	[149]
	In vitro	A549, H322, H460, GLC-82, SPC-A1	↓FBXW7	MiR-544a overexpression/ TINCR knockdown	↑Proliferation; ↑invasion	[150]
	In vitro	PC9, H1299	↓FBXW7	shRNA-mediated silencing	↑EMT	[151]
	In vivo	*FBXW7*+/− mice	↓FBXW7	shRNA-mediated silencing	↑Tumorigenesis	[151]
Oral	In vitro	UM1, UM2, Cal27, SCC1, SCC15, SCC25	↓FBXW7	Binding of miR-24 to the 3-UTR of FBXW7	↑Tumorigenesis	[152]
	In vitro	Tissue samples	↓FBXW7	-	Poor prognosis	[153]
Pancreas	In vitro	BxPC-3, Colo-357	↑FBXW7	Nuclear retention by KPT-185	↓Notch1; ↓ c-Myc; ↓VEGF	[154]
	In vivo	Colo-357 xenografts	↑FBXW7	Nuclear retention by KPT-185	↓Notch1	[154]
	In vitro	MIAPaCa2, BxPC3, PANC1	↓FBXW7	shRNA-mediated silencing	↑β-catenin	[155]
	In vitro	SUIT-2	↓FBXW7	siRNA-mediated silencing	↑MCL-1	[156]
	In vitro	PANC-1, Mia PaCa-2	↑FBXW7	Ectopic overexpression	↑ENT1	[157]
Renal	In vitro	AHCN, A704	↑FBXW7	Ectopic overexpression	↓c-Myc; ↓c-Jun	[158]
	In vitro	786-O, ACHN	↑FBXW7	Ectopic overexpression	↓MMP-2, -9, -13	[159]
Skin	In vitro	MMRU, RPEP	↓FBXW7	siRNA-mediated silencing	↑MAPK/ERK	[15]
	In vivo	*FBXW7*^F/F^ mice	↓FBXW7	-	↑c-Myc	[160]
	In vitro	WC00125, WM39, WM3702, WM3862	↓FBXW7	Nonsynonymous mutations; shRNA-mediated silencing	↑Notch	[161]
	In vivo	Lysm^−^FBXW7^f/f^, Lysm^+^*FBXW7* ^f/f^ mice	↓FBXW7	Myeloid cell-specific deletion	↓MAM	[162]
	In vitro	501mel, SKMEL28, SKMEL24, WM3862, WM39	↓FBXW7	shRNA- mediated silencing	↓Nuclear HSF1	[65]
	In vitro	MM415, MM485, HT144, A2058, SH4	↓FBXW7	siRNA-mediated silencing	↑MITF/PGC-1 signaling	[163]

27-HC: 27-hydroxycholesterol; AURKA: Aurora kinase A; BMSCs: bone marrow-derived stromal cells; CCL2: chemokine (C-C motif) ligand 2; CCNE1: Cyclin-E; CDK2: Cyclin-dependent kinase 2; EglN2: Egl-9 Family Hypoxia Inducible Factor 2; EMT: Epithelial–mesenchymal transition; ENT1: Equilibrative nucleoside transporter 1; ERK: Extracellular signal–regulated kinases; Eya: Eyes absent homolog 1; FAM83D: Family with sequence similarity 83, member D; GRα: Glucocorticoid receptor α; HES1: Hairy and enhancer of split-1; HSF1: Heat-shock factor 1; KLF5: Krueppel-like factor 5; MALAT1: metastasis associated lung adenocarcinoma transcript 1; MAM: metastasis-associated macrophage; MAPK: Mitogen-activated protein kinase; MCL-1: Myeloid cell leukemia 1; MITF: Microphthalmia-associated transcription factor; MMP-2: Matrix metalloproteinase-2; MTDH: Metadherin; PGC-1: Peroxisome proliferator-activated receptor gamma coactivator 1-alpha; PLK: Polo-like kinase; Rictor: Rapamycin-insensitive companion of mTOR; STAT3: Signal transducer and activator of transcription 3; TINCR: Terminal differentiation-induced lncRNA; VEGF: Vascular endothelial growth factor; YAP: Yes-associated protein; ZNF322A: Zinc-finger 322A.

**Table 2:** Role of FBXW7 in cancer cell chemosensitization.

Cancer	Combination	In Vitro/In Vivo	Mechanism of Action	Ref.
Breast	↑FBXW7+ paclitaxel	In vitro	↓MCL-1; ↓PLK-1	[113]
Colorectal	↑FBXW7+ doxorubicin	In vitro	↓EMT	[126]
	↓FBXW7+ irinotecan	In vitro	↑c-Myc; ↓CSC	[203]
CML	↓FBXW7+ imatinib	In vivo	↓LICs	[202]
Gastric	↑FBXW7+ trastuzumab	In vitro	↓MCL-1; ↓c-Myc; ↓c-Jun	[186]
Glioblastoma	↑FBXW7+ temozolomide	In vitro	↓Aurora B; ↓MCL-1; ↓ Notch-1	[106]
Liver	↑FBXW7+ doxorubicin	In vitro	↓EMT	[204]
Lung	↑FBXW7+ cisplatin	In vitro	↓EMT	[205]
Lung	↑FBXW7+ TKI	In vitro	↓MCL-1	[149]
Lung	↑FBXW7+ TKI	In vivo	↓MCL-1	[149]
Nasopharynx	↑FBXW7+ cisplatin	In vitro	↓MRP	[206]
Pancreas	↑FBXW7+ gemcitabine	In vitro	↑ENT1	[157]

CSCs: cancer stem cell; EMT: Epithelial–mesenchymal transition; ENT: equilibrative nucleoside transporter 1; LICs: leukemia-initiating cells; MCL-1: Myeloid cell leukemia 1; MRP: multidrug resistance-associated protein; PLK-1: Polo-like kinase 1; TKI: tyrosine kinase inhibitors.

## Abbreviations

ABC-DLBCL	Activated B-cell like diffuse large B-cell lymphoma
API-1	4-amino-5,8-dihydro-5-oxo-8-β-D-ribofuranosyl-pyrido[2,3-d]pyrimidine-6-carboxamide
ATL	T-cell leukemia
BMSCs	Bone marrow-derived stromal cells
C/EBP-δ	CCAAT/enhancer-binding protein-δ
circRNAs	Circular RNAs
CML	Chronic myeloid leukemia
CPD	Cdc4 phosphodegron
CRC	Colorectal cancer
CSCs	Cancer stem cells
Cul1	Cullin1
DMBA	7,12-dimethylbenz[a]anthracene
DNMT1	DNA methyltransferase 1
EGLN2	Egl-9 Family Hypoxia-Inducible Factor 2
EMT	Epithelial–mesenchymal transition
ENO1	Enolase 1
ENT1	Equilibrative nucleoside transporter 1
EOGC	Early-onset of gastric cancers
ERK	Extracellular signal-regulated kinase
ESCC	Esophageal squamous cell carcinoma
ESCs	Embryonic stem cells
EZH2	Enhancer of zeste homolog 2 polycomb repressive complex 2
FAM83D	Family with sequence similarity 83, member D
FBXL	F-box coupled with LRRs
FBXO	F-box with no motifs
FBXW	F-box coupled with WD repeats
FBXW7	F-box with 7 tandem WD40 repeats
GC	Gastric cancer
GSI	Gamma-secretase inhibitors
GSK3	Glycogen synthase kinase 3
HDAC	Histone deacetylase
Hes-5	Hairy and Enhancer-of-split homologues 5
HIF-1α	Hypoxia-inducible factor-1α
HSF1	Heat-shock factor 1
Hsp90	Heat shock protein 90
IHCC	Intrahepatic cholangiocarcinoma
KLFs	Kruppel-like factors
LICs	Leukemia-initiating cells
LRR	Leucine-rich repeats
MALAT1	Metastasis-associated lung adenocarcinoma transcript 1
MAPK	Mitogen-activated protein kinase
MCL-1	Myeloid cell leukemia 1
MED13	Mediator 13
miR-10b	microRNA-10b
MiRNAs	MicroRNAs
MMP	Metalloproteinase
Mo-MDSCs	Monocytic myeloid-derived suppressor cells
MTDH	Metadherin
mTOR	Mammalian target of rapamycin
NF1	Neurofibromatosis type 1
NF-ΚB	Nuclear factor kappa B
NPC	Nasopharyngeal carcinoma
NRF1	Nuclear factor E2-related factor 1
NSC	Neural stem cell
NSCLC	Non-small cell lung cancer
PC	Pancreatic cancer
PDAC	Pancreatic ductal adenocarcinoma
Pin1	Peptidyl-prolyl cis-trans isomerase NIMA-interacting1
PLK1	Polo-like kinase 1
RCC	Renal cell carcinoma
RhoA	Ras homologue gene family member A
RhoGDI	RHO guanosine diphosphate dissociation inhibitor;
Rictor	Rapamycin-insensitive companion of mTOR
SCF	Skp1-Cullin1-F-box
SCNC	Small cell neuroendocrine carcinoma
SINE	Specific inhibitors of nuclear export
Skp1	S phase kinase-associated protein 1
STAT3	Signal transducer and activator of transcription 3
T-ALL	T-cell acute lymphoblastic leukemia
TAMs	Tumor-associated macrophages
TINCR	Terminal differentiation-induced lncRNA
TNBC	Triple-negative breast cancer
TPA	12-O-tetradecanoylphorbol-13-acetate
Ubc	Ubiquitin-conjugating enzyme
UPS	Ubiquitin-proteasome system
WT	Wilms’ tumor
YAP	Yes-associated protein
ZNF322A	Zinc-finger 322A
